# Potassium Channels in the Transition from Fetal to the Neonatal Pulmonary Circulation

**DOI:** 10.3390/ijms23094681

**Published:** 2022-04-23

**Authors:** Chandran Nagaraj, Yingji Li, Bi Tang, Natalie Bordag, Divya Guntur, Péter Enyedi, Horst Olschewski, Andrea Olschewski

**Affiliations:** 1 Ludwig Boltzmann Institute for Lung Vascular Research, Neue Stiftingtalstraße 6, 8010 Graz, Austria; nagaraj.chandran@lvr.lbg.ac.at (C.N.); natalie.bordag@lvr.lbg.ac.at (N.B.); 2 Experimental Anaesthesiology, Department of Anaesthesiology and Intensive Care Medicine, Medical University of Graz, Auenbruggerplatz 5, 8036 Graz, Austria; liy@ice-biosci.com (Y.L.); bitang2000@163.com (B.T.); divya.guntur@medunigraz.at (D.G.); 3 Department of Dermatology and Venereology, Medical University of Graz, Auenbruggerplatz 8, 8036 Graz, Austria; 4 Department of Physiology, Faculty of Medicine, Semmelweis University, Tűzoltó utca 37-47, 1094 Budapest, Hungary; enyedi.peter@med.semmelweis-univ.hu; 5 Division of Pulmonology, Department of Internal Medicine, Medical University of Graz, Auenbruggerplatz 15, 8036 Graz, Austria; horst.olschewski@medunigraz.at

**Keywords:** fetal circulation, smooth muscle cells, pulmonary artery, ductus arteriosus, TASK-1, pH-sensitivity, patch-clamp

## Abstract

The transition from the fetal to the neonatal circulation includes dilatation of the pulmonary arteries (PA) and closure of the Ductus Arteriosus Botalli (DAB). The resting membrane potential and various potassium channel activities in smooth muscle cells (SMC) from fetal and neonatal PA and DAB obtained from the same species has not been systematically analyzed. The key issue addressed in this paper is how the resting membrane potential and the whole-cell potassium current (IK) change when PASMC or DABSMC are transitioned from hypoxia, reflecting the fetal state, to normoxia, reflecting the post-partal state. Patch-clamp measurements were employed to characterize whole-cell K^+^ channel activity in fetal and post-partal (newborn) PASMC and DABSMC. The main finding of this paper is that the SMC from both tissues use a similar set of K^+^ channels (voltage-dependent (Kv), calcium-sensitive (KCa), TASK-1 and probably also TASK-2 channels); however, their activity level depends on the cell type and the oxygen level. Furthermore, we provide the first evidence for pH-sensitive non-inactivating K^+^ current in newborn DABSMC and PASMC, suggesting physiologically relevant TASK-1 and TASK-2 channel activity, the latter particularly in the Ductus Arteriosus Botalli.

## 1. Introduction

In the newborn, with the very first breath, the function of gas exchange is transferred from the placenta to the lungs, and the pulmonary circulation takes over the circulation from the placenta. The transition from the fetal to the neonatal circulation includes lung expansion, an increase of pulmonary arterial (PA) blood flow to accommodate the entire cardiac output and closure of the Ductus Arteriosus Botalli (DAB). Accordingly, the intrauterine to extrauterine circulation adaptation is a remarkably complex physiological process and requires a concerted but opposite action in the pulmonary arteries and the ductus arteriosus. The pulmonary vasculature dilates immediately, whereas the fetal extracardiac shunt pathways, including the ductus arteriosus, constrict and close gradually. These physiological alterations are mandatory for normal neonatal life.

The mechanisms that mediate the postnatal fall in PA resistance and closure of the DAB are not yet fully elucidated. Still, it is well accepted that the change in the tone of the smooth muscle cells in both vessels contributes significantly to this response [1,2]. The smooth muscle cells’ vascular tone strongly depends on cell membrane potassium channel activity which determines the cells’ resting membrane potential (Em) [3,4]. Increased activity of the potassium channels causes hyperpolarization of the SMC, inhibition of the voltage-gated Ca^2+^ channels and a decrease in cytosolic calcium, which results in vasodilatation. Inhibition of these channels leads to depolarization with a subsequent increase in cytosolic calcium concentrations, resulting in vasoconstriction [5]. In both fetal PASMC and DABSMC, calcium-dependent (K_Ca_) and several voltage-sensitive (K_v_) potassium (K^+^) channels have been identified over the past decades. Functional investigations of these channels revealed that their activity may depend on changes in oxygen tension or on the redox state [6]. In fetal lambs, activation of K_Ca_ via a cyclic nucleotide-dependent kinase was reported to drive the oxygen-induced fetal pulmonary vasodilation [7,8]. In the human DAB, K_v_ channels have been reported to modulate vessel tone [9,10]. Other potassium channel family members, such as ATP- dependent (K_ATP_), inward-rectifying or non-inactivating two-pore domain K^+^ channel current (e.g., TASK-1) have been detected mainly in adult PASMC [11,12,13].

Our study aimed to systematically compare potassium channel currents and resting membrane potential of primary smooth muscle cells isolated from rat PA and DAB under fetal and postnatal conditions. Detailed investigations of the acute and chronic changes occurring during the transition from fetal to newborn circulation are essential to better understand this fundamental process in the life of every mammal.

## 2. Results

**Figure 1:**
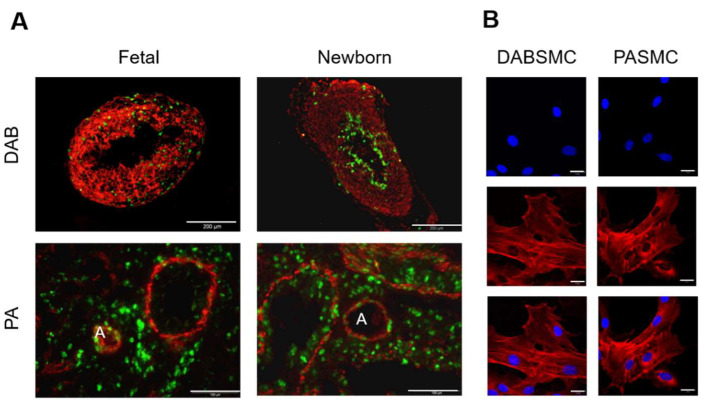
DAB and PA obtained from fetal and newborn rats. (**A**) Representative images of smooth-muscle α-actin (red) and Ki67 protein (green) expression detected by immunofluorescence in DAB (upper panel) and PA (lower panel) obtained from fetal and newborn rats. Scale bar = 200 μm for DAB and 100 µM for lung slices showing PAs indicated by A (artery). (**B**) Fluorescent immunostainings indicate DAPI nuclear staining (blue), smooth-muscle α-actin (red) and an overlayed image (merged) in a single–plane confocal image of DABSMC and PASMC. Scale bar = 20 μm.

To directly compare the SMC’ electrophysiological properties obtained from DAB and PA of fetal rats, we established the preparation and maintenance of both tissues from the same animals in an uninterrupted hypoxic environment. Using the XVIVO hypoxia workstation, the pO_2_ was constantly monitored and kept between 34 and 38 mmHg (HOX; app. 4.5–5% O_2_). This environment mimics the fetal conditions for PA and DAB. In contrast, the tissue from the two-day-old newborn animals (referred to as newborn rats throughout) was prepared and maintained under normoxia (pO_2_ between 140 and 159 mmHg; app. 18.5–21% O_2_). The tissue was stained and the representative images show the immunofluorescent staining for α-smooth muscle actin and Ki 67 on DAB and lung sections with PAs from fetal and newborn rats (Figure 1A). Ki67 staining was used to detect proliferation in the tissue sections. The constricted DAB and expanded PA from newborns are displayed in the right column. α-smooth muscle actin is one of the markers for vascular SMC. The representative image in Figure 1B shows the fetal DABSMC and PASMC used for our studies.

### 2.1. Resting Membrane Potential (Em) and Pharmacology of the Whole-Cell K^+^ Current (IK) in Fetal Rat DABSMC

**Figure 2:**
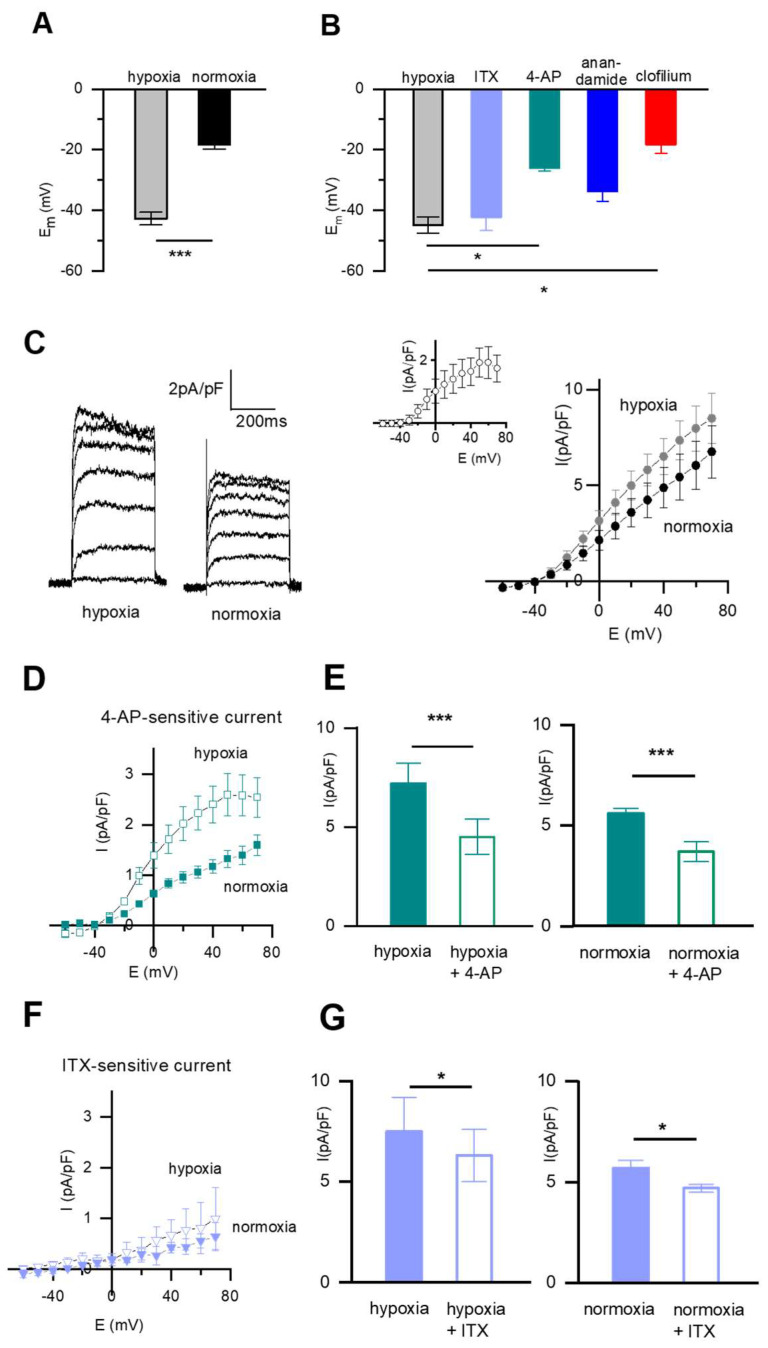
Pharmacology of the resting membrane potential and the whole-cell K^+^ current in fetal rat DABSMC under hypoxia. (**A**) Modulation of the DABSMC resting membrane potential by acute normoxia (*n* = 22, N ≥ 10 in each group). (**B**) Average membrane potentials recorded in current-clamp configuration under hypoxic conditions and after exposure to either ITX, or 4-AP, or anandamide or clofilium (*n* ≥ 6 in each group and N ≥ 4 in each group). (**C**) Modulation of the whole cell K+ current by acute normoxia. Representative traces recorded from DABSMC during hypoxia and after exposure to acute normoxia (left). SMC were voltage clamped at a holding potential of −60 mV, and +10 mV steps evoked currents to more positive potentials (+70 mV) with test pulses of 400-ms duration. Average current density-voltage plot showing current densities (in pA/pF) recorded from single DABSMC (right). Insert shows the normoxia-sensitive current density. (*n* = 6, and N ≥ 4 in each group). (**D**) Average current density-voltage plot showing 4-AP-sensitive current densities recorded in hypoxia or after exposure to acute normoxia from single DABSMC (*n* = 6, and N ≥ 5 in each group). (**E**) Average current density showing effects of 4-AP at +70 mV in hypoxia (left) and in normoxia (right). (*n* = 6, and N ≥ 5 in each group). (F) Average current density-voltage plot showing ITX-sensitive current densities recorded in hypoxia or after exposure to acute normoxia from single DABSMC. (*n* = 6, and N ≥ 5 in each group). (**G**) Average current density showing effects of ITX at +70 mV in hypoxia (left) and in normoxia (right). (*n* = 6, and N ≥ 5 in each group). Values are mean ± S.E.M. * *p* < 0.05, *** *p* < 0.01 difference from control (hypoxia in **A**, **B**, **E** and **G** (left columns) and from normoxia in **E** and **G** right columns).

First, we focused on the resting membrane potential, as this is substantially driven by IK in SMC and investigated the effects of different inhibitors on the Em. In *n* = 22 native fetal DABSMC in hypoxia, the average resting membrane potential was −42.7 ± 0.8 mV. After 10 min exposure to normoxia, the cells depolarized to −18.3 ± 1.5 mV (*n* = 22; *p* < 0.001; Figure 2A). In an independent setting, alterations of Em were induced by different inhibitors. Figure 2B shows changes in Em in response to 5 mM 4-AP (K_v_ channel blocker), 100 nM ITX (K_Ca_ channel inhibitor), 10 µM anandamide (TASK-1 inhibitor) and 100 µM clofilium (TASK-2 inhibitor). These blockers were applied to *n* = 6 cells. The Em of these cells at baseline was −44.8 ± 3.8 mV. The K_v_ channel inhibitor 4-AP depolarized the cells to −26.0 ± 0.9 mV (*p* < 0.05), and clofilium, the TASK-2 blocker to −18.3 ± 2.9 mV (*p* < 0.05). The Em was not significantly affected by ITX. The depolarizing effect of anandamide was moderate (to −33.8 mV) without reaching statistically significant. This suggests that the highly negative resting membrane potential of hypoxic DABSMC is mainly driven by TASK-2 and K_v_ channel activity, TASK-1 may contribute whereas K_Ca_ channels do not contribute to the Em.

Basal whole-cell outward potassium currents (I_K_) in fetal DABSMC were recorded from holding potentials of –60 mV using a perforated patch-clamp technique in hypoxia and after exposure to normoxia. Under these conditions, normoxia rapidly reduced I_K_, as shown in Figure 2C. The current-voltage relationship for I_K_ illustrating the change from normoxia to hypoxia is shown in Figure 2C (right panel). The inset displays the normoxia-sensitive current. The whole-cell current was sensitive to inhibition by 5 mM 4-AP, less pronounced and mainly at higher potentials also to 100 nM ITX both under hypoxic and normoxic conditions. Figure 2D shows average current density-voltage plots of 4-AP-sensitive and ITX-sensitive current densities. During hypoxia, the 4-AP sensitive current was significantly larger than during normoxia (*p* < 0.05 at 0 mV). The non-inactivating current (I_KN_) carried out by background K^+^ channels e.g. TASK-1 and TASK-2 were evaluated in a separate setting. The results will be shown in Figure 4.

### 2.2. The Resting Membrane Potential (Em) and the Pharmacology of the Whole-Cell K^+^ Current in Fetal Rat PASMC

**Figure 3:**
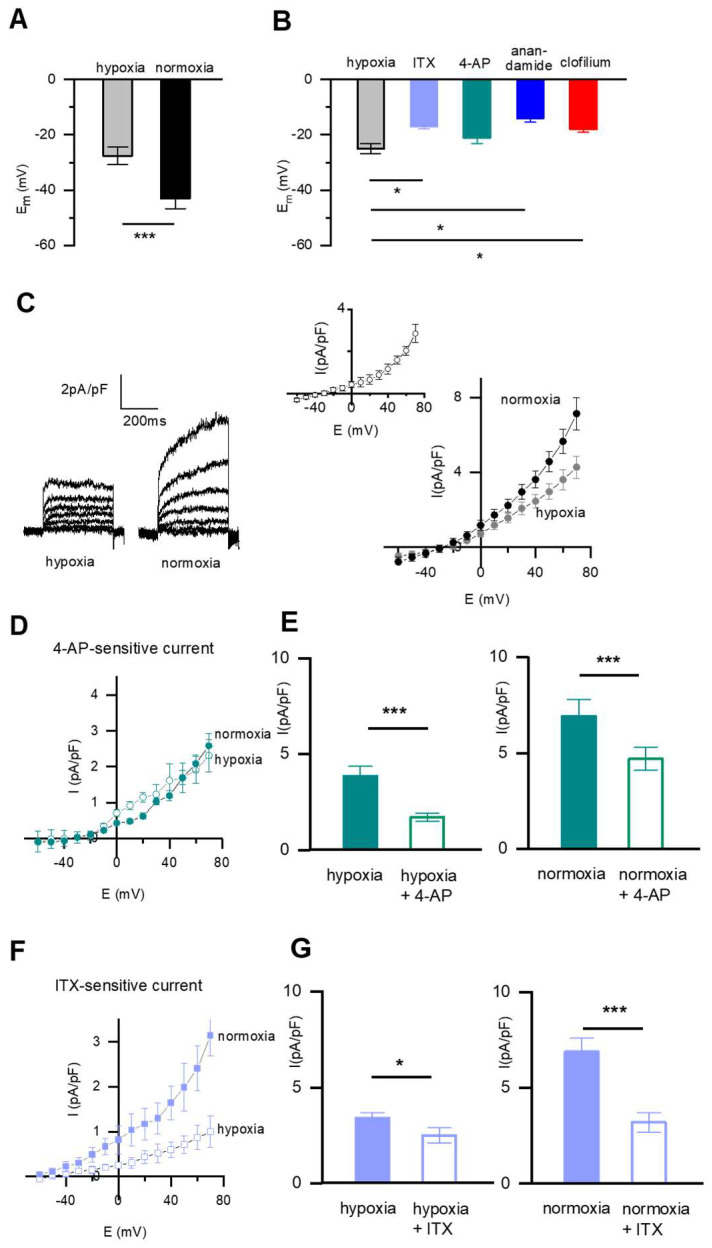
Pharmacology of the resting membrane potential and the whole-cell K^+^ current in fetal rat PASMC under hypoxia. (**A**) Modulation of the PASMC resting membrane potential by acute normoxia (*n* = 24, N ≥ 10 in each group). (**B**) Average membrane potentials recorded in current-clamp configuration under hypoxic conditions and after exposure to either ITX, or 4-AP, or anandamide or clofilium (*n* ≥ 6 in each group and N ≥ 3 in each group). (**C**) Modulation of the whole cell K^+^ current by acute normoxia. Representative traces recorded from PASMC during hypoxia and after exposure to acute normoxia (left). SMC were voltage clamped at a holding potential of −60 mV, and +10 mV steps evoked currents to more positive potentials (+70 mV) with test pulses of 400-ms duration. Average current density-voltage plot showing current densities (in pA/pF) recorded from single PASMC (right). Insert shows the normoxia-sensitive current density. (*n* = 6, and N ≥ 4 in each group). (**D**) Average current density-voltage plot showing 4-AP-sensitive current densities recorded in hypoxia or after exposure to acute normoxia from single PASMC (*n* = 6, and N ≥ 3 in each group). I Average current density showing effects of 4-AP at +70 mV in hypoxia (left) and in normoxia (right). (*n* = 6, and N ≥ 3 in each group). (**F**) Average current density-voltage plot showing ITX-sensitive current densities recorded in hypoxia or after exposure to acute normoxia from single PASMC. (*n* = 6, and N ≥ 3 in each group). (**G**) Average current density showing effects of ITX at +70 mV in hypoxia (left) and in normoxia (right). (*n* = 6, and N ≥ 3 in each group). Values are mean ± S.E.M. * *p* < 0.05, *** *p* < 0.01 difference from control (hypoxia in **A**, **B**, and **E** and **G** (left columns) and from normoxia in **E** and **G** right columns).

As depicted in Figure 3, in *n* = 24 fetal PASMC in hypoxia, the Em was −27.6 ± 1.8 mV. Ten minutes exposure of the cells to normoxia caused a significant hyperpolarizing shift of the Em to −42.9 ± 2.1 mV (*p* < 0.001; Figure 3A), i.e., the normoxic milieu caused a change in the opposite direction than that observed in DABSMC. The effect of various K^+^ channel inhibitors on Em was tested under hypoxic conditions in an independent setting in *n* = 6 PASMC. The Em at baseline was −24.8 ± 1.2 mV and was further depolarized by 100 nM ITX to −17.0 ± 0.6 mV (*p* < 0.05), by 10 µM anandamide to −13.2 ± 1.5 mV (*p* < 0.05) and by clofilium to −17.6 ± 1.0 mV (*p* < 0.05) (Figure 3B), but not by 5 mM 4-AP (data not shown), suggesting that K_Ca_ and TASK-1 and TASK-2 were active around the Em in hypoxia but K_v_ channels were not.

Basal whole-cell outward Ik in fetal PASMC was recorded from holding potentials of −70 mV using a perforated patch-clamp technique in hypoxia and after exposure to normoxia. Under these conditions, normoxia rapidly increased I_K_, as shown in Figure 3C. The current-voltage relationship for I_K_ illustrating the change from hypoxia to normoxia is shown in Figure 3C (right panel). The sensitivity to 4-AP did not change from hypoxia to normoxia, suggesting that the 4-AP-sensitive current was not oxygen sensitive in contrast to the ITX-sensitive current (Figure 3D).

### 2.3. pH-Sensitive Em and Current Is Present in SMC from DAB and PA

**Figure 4:**
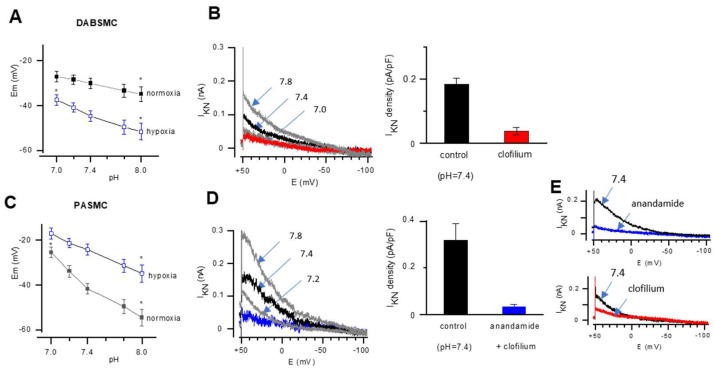
Presence of a pH-sensitive current in DABSMC and PASMC. (**A**) Modulation of the resting membrane potential by pH. Average resting membrane potentials recorded under hypoxic conditions in fetal rat DABSMC and in normoxia in newborn rat DAB-SMC (*n* ≥ 5 in each group, N ≥ 3 in each group). (**B**) Representative traces recorded from DABSMC of a newborn rat in normoxia showing the modulation of I_KN_ by pH or clofilium (red line) (left) and the average current density calculated at 0 mV (right) (*n* ≥ 5 in each group, N ≥ 3 in each group). Non-inactivating potassium current was obtained from a holding potential of 0 mV. Then the voltage was stepped to 60 mV and further ramped to −110 mV over a period of 1.6 s. (**C**) Average resting membrane potentials recorded under hypoxic conditions in fetal rat PASMC in hypoxia and newborn rat PASMC in normoxia shows the modulation of the resting membrane potential by pH. (*n* ≥ 5 in each group, N ≥ 3 in each group). (**D**) Representative traces recorded from PASMC of a newborn rat in normoxia showing the modulation of I_KN_ by pH or clofilium and anandamide (blue line) (left) and the average current density calculated at 0 mV (*n* ≥ 5 in each group) (right). (**E**) Representative traces recorded from PASMC of a newborn rat in normoxia showing the effect of anandamide (blue line) and clofilium (red line) at pH = 7.4. Values are mean ± S.E.M. * *p* < 0.05 difference from control (pH = 7.4).

As two-pore-domain channels like TASK-1 or TASK-2 are known to be pH-sensitive [14], we tested the effect of pH changes on Em and the non-inactivating whole cell K^+^ current (IKN). The Em of SMC from both tissues were sensitive to extracellular pH changes over a broad range between pH = 7.0 and 8.0 (Figure 4A). Acidosis (pH = 7.0) depolarized hypoxic DABSMC to −37.5 ± 2.3 mV (*n* = 5) and normoxic PASMC to −25.5 ± 2.3 mV (*n* = 5). When the pH was increased to 8.0, significant hyperpolarization was detected in the cells under both conditions (hypoxic DABSMC to −51.7 ± 3.7 mV (*n* = 5) hypoxic PASMC to −34.9 ± 3.9 mV (*n* = 5); normoxic DABSMC to −34.9 ± 3.3 mV (*n* = 5) and normoxic PASMC to −54.6 ± 3.6 mV (*n* = 5).

Next, we investigated IKN in DABSMC and PASMC of newborn rats under normoxic conditions. In DABSMC, the I_KN_ was small, but sensitive to changes in extracellular pH. 100 µM clofilium strongly inhibited I_KN_, suggesting that the main source of I_KN_ was TASK-2. Similarly, in PASMC, acidosis inhibited and alkalosis enhanced I_KN_. Although anadamide and clofilium reduced IKN, the strongest effect could be detected when the inhibitors were applied together. These observations suggest that the current is due to both TASK-1/TASK-2 channels (Figure 4B,C).

### 2.4. Resting Membrane Potential and Whole-Cell K^+^ Current in Newborn Rat DABSMC and PASMC

**Figure 5:**
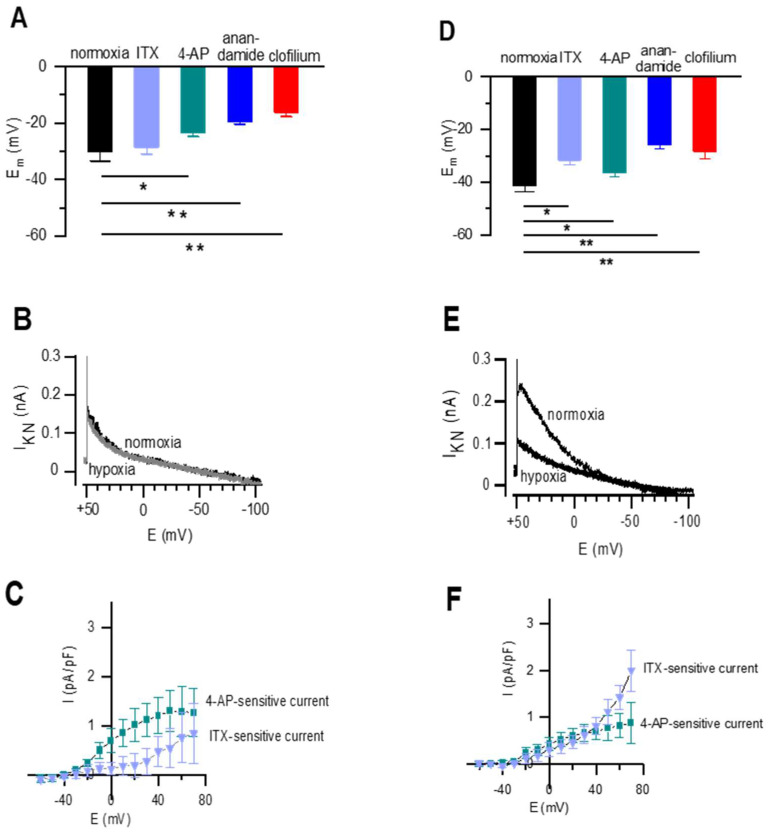
Resting membrane potential and pharmacology of the whole-cell K^+^ current in newborn rat DABSMC and PASMC in normoxia. (**A**) Average membrane potentials of newborn rat DABSMC recorded under normoxic conditions and after exposure to either ITX, or 4-AP, or anandamide or clofilium (*n* ≥ 6 and N ≥ 3 in each group). (**B**) Representative traces of I_KN_ recorded in newborn rat DABSMC under normoxic conditions showing the lack of the effect of acute hypoxia. I_KN_ was obtained from a holding potential of 0 mV. Then the voltage was stepped to +60 mV and further ramped to −100 mv over a period of 1.6 s. (**C**) Average current density-voltage plot showing 4-AP-sensitive or ITX-sensitive current densities in DABSMC. (*n* = 6 and N = 3 in each group). (**D**) Average membrane potentials of newborn rat PASMC recorded under normoxic conditions and after exposure to either ITX, or 4-AP, or anandamide or clofilium (*n* ≥ 6 and and N = 3 in each group). (**E**) Representative traces of IKN recorded in newborn rat PASMC under normoxic conditions showing the effect of acute hypoxia. I_KN_ was obtained from a holding potential of 0 mV. Then the voltage was stepped to +60 mV and further ramped to −100 mv over a period of 1.6 s. (**F**) Average current density-voltage plot showing 4-AP-sensitive or ITX-sensitive current densities in newborn rat PASMC in normoxia (*n* = 6 and N = 3 in each group). Values are mean ± S.E.M. * *p* < 0.05 and ** *p* < 0.01 difference from control (normoxia).

The average resting Em from newborn DABSMC in normoxia was −30.0 ± 3.4 mV (*n* = 5). Figure 5A shows the effects of 4-AP, ITX, anandamide and clofilium in these cells. In constant normoxic environment, 4-AP depolarized the cells to −23.3 ± 1.5 mV, anandamide to −19.5 ± 0.9 mV and clofilium to −16.2 ± 2.6 mV (*p* < 0.05, respectively), but ITX had no significant effect, indicating that K_Ca_ channels did not contribute to the membrane potential. Since pH-sensitive I_KN_ was detected in postnatal DABSMC, we recorded I_KN_ under normoxic and acute hypoxic conditions. As shown in Figure 5B, I_KN_ was not oxygen sensitive. In contrast, the whole potassium current was strongly sensitive to 4-AP. The 4-AP and ITX-sensitive currents are shown in Figure 5C.

The average resting Em of post-partal PASMC in normoxia was −41.2 ± 1.2 mV (*n* = 5). Figure 5D shows the effects of 4-AP, ITX, anandamide and clofilium on Em. All antagonists depolarized the resting membrane potential significantly. ITX to −31.5 ± 1.9 mV (*p* < 0.05), 4-AP to −36.2 ± 1.5 mV (*p* < 0.05), anandamide to −25.7 ± 1.8 mV (*p* < 0.01) and clofilium to −28.2 ± 2.8 mV (*p* < 0.01). In contrast to DABSMC, I_KN_ was strongly inhibited by hypoxia as shown in Figure 5E. Finally, I_K_ was inhibited by both 4-AP and ITX. The 4-AP and ITX-sensitive currents are shown in Figure 5F, suggesting that both K_v_ and K_Ca_ channels were present.

## 3. Discussion

Investigation of the resting membrane potential and various K^+^ channel activities in SMC from fetal and neonatal pulmonary arteries and ductus arteriosus obtained from the same animals has not been systematically analyzed. Therefore, the key issue addressed in the current paper is how the resting membrane potentials and the whole-cell K^+^ currents change when PASMC or DABSMC are exposed to various oxygen tensions or treated with different K^+^ channel inhibitors. For this study, we developed a method to isolate and maintain SMC from both vessel types under controlled oxygen tensions mimicking the fetal and postnatal conditions and characterized the different SMC and the changes of their conductance during the transition. The main finding of the current paper is that the SMC from both tissues use virtually similar K^+^ channels, but oxygen has opposite effects.

**Figure 6:**
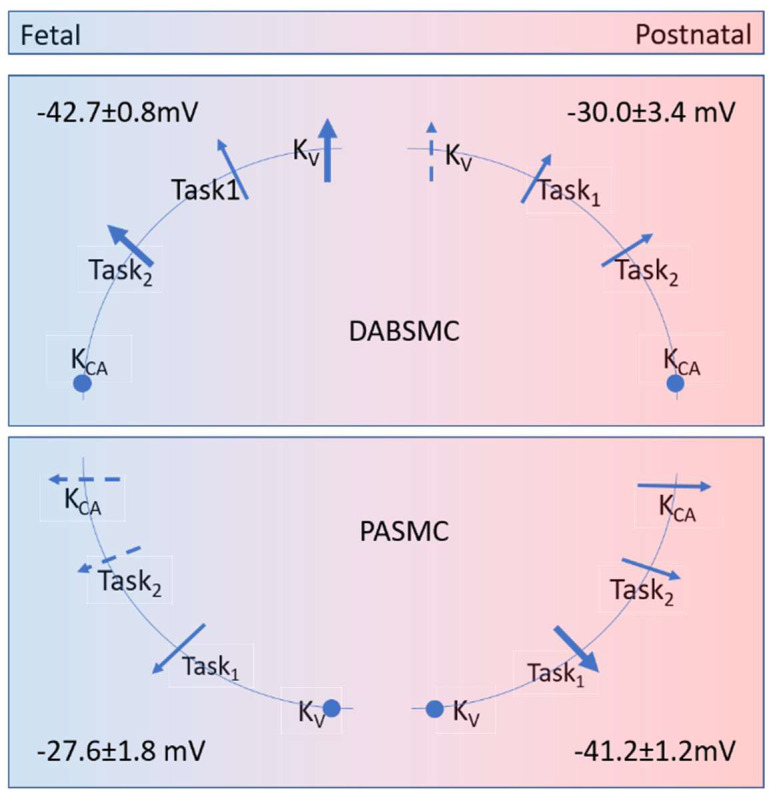
Graphical summary. Membrane potential (Em) of Ductus Arteriosus Botalli smooth muscle cells (DABSMC) and pulmonary arterial smooth muscle cells (PASMC) in the fetal (hypoxic) and postnatal (normoxic) state. The arrows indicate the functional contribution of different potassium channels, based on the effects of specific inhibitors as tested in 5 or 6 cells, respectively. For Kv, TASK-1, TASK-2 and Kca channels, the specific blockers 4-aminopyidine, anandamide, clofilium, and iberiotoxin were used. Thick arrows: Em-effect > 15 mV; thin arrows, Em-effect 9–15 mV; dashed arrows, Em-effects 5–9 mV; dots, Em-effects ≤ 5 mV.

Based on their pharmacology, the potassium channels can be classified into voltage-dependent (K_v_), calcium-sensitive (K_Ca_) and K_2P_ channels in the pulmonary circulation. For the first time, we detected hypoxia-sensitive I_KN_ in newborn PASMC and strongly pH-sensitive I_KN_ in DABSMC and PASMC of newborn rats. The source of the latter is very likely the K_2P_ channels TASK-1 and TASK-2. Our proposed overview of the main contributing K^+^ channels is given in Figure 6.

Describing phenotypes of SMC from pulmonary arteries and the ductus arteriosus is of interest since these cells play a crucial role in evoking the vascular changes at birth. During the transition from intra- to extrauterine life, the pulmonary vascular resistance instantly falls from a very high fetal level to a low level. This process allows pulmonary blood flow to increase 8- to 10-fold and enables the lung to fulfill its postnatal role in gas exchange [15]. Parallel to the decrease in pulmonary vascular resistance, the ductus arteriosus starts to constrict, first functionally and later anatomically. Consequently, the shunt of venous blood to the descending aorta closes under physiological conditions [2]. The factors that contribute to these processes include increased oxygen tension, mechanical distension of the lung by ventilation, shear stress, enhanced production of vasodilator and vasoconstrictor substances and metabolic changes [16,17,18,19]. The transition requires immediate and well-orchestrated steps. Accordingly, we observed the changes in K^+^ channel function in the smooth muscle cells of both vessels.

Many studies have reported that changes in oxygen tension influence various K^+^ currents including K_v_, K_Ca_, K_ATP_ or TASK-1 in the systemic and pulmonary circulation at different locations and in different species [7,20,21,22,23,24,25,26,27,28]. First, we focused on the resting membrane potential and its change from hypoxia to normoxia. Our current-clamp experiments with the different inhibitors suggest that different K^+^ channels are simultaneously active in both tissues in both hypoxia and normoxia. In hypoxia, in DABSMC, the K_v_ and the TASK-2 blockers exhibited significant depolarization, whereas in PASMC the membrane potential was already depolarized. Nevertheless, TASK-1 inhibition further depolarized Em, suggesting some TASK-1 activity under these conditions. The K_Ca_ and the TASK-2 inhibitor had a small effect, while the K_v_ blocker had no significant effect. The postpartal PASMC under normoxia had a significantly higher Em, to which TASK1 channels contributed strongly, but also TASK-2 and K_Ca_ channels, while K_v_ channels did not (Figure 6). The contribution of so many channels to Em in the different cells and conditions is surprising, as previous reports usually highlighted the role of only one specific K^+^ channel in any of these SMC. On the other hand, the presence of multiple channels has never been excluded.

The effects of the different inhibitors on the whole cell current in both tissues under both conditions were in line with the effects on Em. In DABSMC, the 4-AP sensitive current was strongly oxygen-sensitive, whereas the ITX sensitive current was not. This suggests that K_v_ channels are strongly activated under hypoxic conditions and inhibited by normoxia but the K_Ca_ channels are not. These findings are in line with previous studies [9,29]. In PASMC, the 4-AP sensitive current was not oxygen dependent, while the ITX-sensitive current was strongly activated by oxygen. This suggests that in PASMC, K_Ca_ are activated by oxygen but not K_v_ channels.

The K_2P_ channel family has gained significant interest in the cardiovascular system during the last decade [30]. K_2P_ are involved in the complex regulation of diverse physiological functions and underlie the leak conductance that is central in determining the resting membrane potential of many cells. In our study, we showed for the first time, that K_2P_ channels contribute to the resting membrane potential of fetal and newborn DABSMC and PASMC. In fact, we detected impressive pH sensitivity of the resting membrane potential in both cell types obtained from both fetal and newborn animals. It is important to note that TASK-1 shares the extracellular pH sensitivity with TASK-2 although at a different pH range [31]. Furthermore, hypoxia-sensitive IKN was seen in newborn PASMC, a characteristic feature of TASK-1 and TASK-2. Together with the effects of the specific blockers on the resting membrane potential, we can conclude that in DABSMC at least TASK-2 and in PASMC TASK-1 and TASK-2 are functionally relevant. Because fetal lung is not perfused and the fetal lung fluid is normally acidic, with a pH of 6.3 [32], our data would demonstrate the contribution of the pH-sensitivity to the hyperpolarization of PASMC, which contributes to the vasodilation and thus, to the tremendous pulmonary vascular resistance drop after birth, when the pH shifts towards normal. Our findings are in line with previous reports showing that the TASK-1 channel is a significant component of the I_KN_ background current and contributes to the resting membrane potential, vasomotor tone, and proliferation of PASMC obtained from adults [12,24,33]. In addition, our data suggest an important contribution of TASK-2, especially in newborn animals.

In conclusion, this study characterized whole-cell K^+^ channel activity in SMC from fetal and neonatal pulmonary arteries and ductus arteriosus. We found that SMC in both tissues display a mixed phenotype implicating the role of different K^+^ channels in controlling both fetal and neonatal vascular resistance. Furthermore, we provide the first direct evidence for pH-sensitive, non-inactivating K^+^ current in newborn DABSMC and PASMC suggesting physiologically relevant TASK-1 and TASK-2 channel activity, the latter particularly in the Ductus Arteriosus Botalli.

## 4. Materials and Methods

The adult rats were killed by rapid cervical dislocation under deep CO_2_ anaesthesia and fetal pups and two-days-old newborn rats (referred as newborn rats throughout) were killed by rapid decapitation according to the German and Austrian guideline standards. The experimental procedure was in full accordance with national and institutional guidelines.

### 4.1. Cell Isolation

Primary smooth muscle cells (SMC) were isolated from resistance pulmonary arteries and from the ductus arteriosus [34,35]. The adventitia was carefully removed under stereomicroscopic guidance and media pieces <1 mm^3^ were placed onto 16-mm coverslips with 500 μL culture medium (Promocell Medium supplemented with penicillin and gentamicin; Promocell, Heidelberg, Germany). Cells were maintained at 37 °C; the medium was initially changed after 24 h, and then every 48 h. SMC grown on coverslips were used for patch-clamp recordings within 6 days. SMC identity was verified by their characteristic appearance in phase-contrast microscopy. The purity of SMC cultures was confirmed using indirect immunofluorescent antibody staining for SM-specific isoforms of α-actin and myosin (at least 95% of cells stained positive), and lack of staining for von Willebrand factor.

The preparation of fetal SMC was carried out in hypoxic bags using integrated gas controllers. The cells were maintained in the XVIVO hypoxic workstation from Biospherix (BioSpherix, Lacona, NY, USA) using fully integrated incubators and work station with dynamic programmable hypoxic and non-condensing humidity control at 37 °C. Solutions used in these studies were equilibrated and kept in the hypoxic workstation.

### 4.2. Current Recording and Analysis

Whole-cell recordings were performed using the amphotericin-perforated patch-clamp technique as described previously [23,36,37,38]. Patch pipettes were pulled from glass tubes (PG 150T, Warner Instruments Corp, Holliston, MA, USA). The pipettes were fire-polished directly before the experiments and had a resistance of 2–3 MΩ when filled with a pipette solution. In all voltage- and current-clamp experiments, the patch-clamp amplifiers were Axopatch 200A and B (Molecular Devices, San Jose, CA, USA). Offset potentials were nulled directly before the formation of the seal. Capacitance was corrected for, and perforation was monitored by changes in series resistance. Capacitance was detected before the start and at the end of the recordings. Cells developing more than 10% changes were excluded. The small currents recorded in the fetal and newborn cells made series resistance compensation unnecessary [39]. The control (hypoxic or normoxic) voltage-clamp recordings have been carried out after 4 min in the bath solution and the effects of the inhibitors have been recorded after another 4 min in the bath solution containing the blocker [23]. SMC were voltage clamped at a holding potential of −60 mV, and +10 mV steps evoked currents to more positive potentials (+70 mV) with test pulses of 400-ms duration. Steady-state current-voltage relationships were obtained by measuring the current at the end of the voltage-clamp pulse. Non-inactivating potassium current was obtained from a holding potential of 0 mV. Then the voltage was stepped to 60 mV and further ramped to −110 mV over a period of 1.6 s. In order to isolate the non-inactivating potassium current from other voltage-dependent potassium currents, cells were clamped at 0 mV for at least 5 min. As we have previously shown, under these conditions’ inhibitors of K_Ca_ or K_v_ channels have no significant effect on the non-inactivating potassium current [24,35]. The effective corner frequency of the low-pass filter was 1 kHz. The frequency of digitization was at least twice that of the filter. Cells were held in current clamp for resting membrane potential (Em) experiments at their resting Em (without current injection). Stable Em values can be usually detected after app. 15 min.

The data were stored and analyzed with commercially available pCLAMP software (Molecular Devices, San Jose, CA, USA). All experiments were performed at 30 °C and in low light intensity because of the light sensitivity of amphotericin B.

### 4.3. Solutions

Cells were superfused with bath solution of the following composition (in mmol/L): 140.5 NaCl, 5.5 KCl, 1.5 CaCl_2_, MgCl_2_ 1, glucose 10, 0.5 Na_2_HPO_4_, 0.5 KH_2_PO_4_, HEPES 10; adjusted to pH 7.3 with NaOH. The standard intracellular pipette solution contained (in mM) 145 KCl, 1 MgCl_2_, 1 K_2_ATP, 0.1 EGTA, 10 HEPES 10, and 120 μg/mL amphotericin B (pH was adjusted to 7.2 by KOH). K_2_ATP was removed for the investigations of KATP [40].

All compounds were purchased from Sigma (St. Louis, MO, USA). Antagonists were directly added to the control (bath) solutions (diluent control), applied immediately before the start of the experiments and the steady-state effects were recorded and calculated. The pH of solutions containing drugs was tested and corrected to eliminate potential pH-induced effects.

The effect of hypoxia (HOX) and normoxia (NOX) was studied by switching between normoxic and hypoxic or between hypoxic and normoxic perfusate reservoirs, respectively. Normoxic solutions were equilibrated with 21% O_2_ plus balance N_2_. Hypoxic solutions were achieved by bubbling with 5% O_2_ (plus balance N_2_) for at least 20 min before cell perfusion and by blowing N_2_ over the surface of the experimental chamber using a modified dish [23]. These procedures produced pO_2_ values in the experimental chamber of 140–160 mmHg (21% O_2_) and 35–42 mmHg (5% O_2_). O_2_ levels were measured with a Rapidlab Chiron blood gas analyzer from samples taken directly from the experimental chamber containing the SMC during perfusion, which allows an exact measurement of pO_2_. By the use of a small recording chamber (400 μL), high perfusion rate (2–3 mL/min), and short dead space, bath exchange could be achieved in <30 s.

### 4.4. Immunofluorescence STAINING

Immunofluorescence was performed as previously described [33].

### 4.5. Statistical Analysis

Numerical values are given as means ± SEM (standard error of the mean) of n cells. N indicates the number of the animals and n indicates the number of the cells. Intergroup differences were assessed by a factorial analysis of variance with post hoc analysis with Fisher’s least-significant difference test or Student’s unpaired and paired t tests as appropriate. Probability values of <0.05 were considered significant.

## Data Availability

Not applicable.
